# Aortic dissection in pregnancy

**DOI:** 10.11604/pamj.2021.38.406.29421

**Published:** 2021-04-28

**Authors:** Fares Ben Mansour, Hassen Ibn Hadj Amor

**Affiliations:** 1 Department of Cardiology, Taher Sfar Hospital, Mahdia, Tunisia

**Keywords:** Annulo-aortic ectasia, aortic dissection, pregnancy

## Image in medicine

A 36-year-old pregnant woman gravida parity abortus (G3P2A1) at 36 weeks of gestation with no family history of sudden death or aortic dissection consulting several times for chest pain and dyspnea for 8 days, wrongly blamed on pregnancy. Faced with the persistence of symptoms, she consulted the emergency room. The patient stated that she had breathing discomfort occurring within 8 hours. Physical examination revealed: right and left non-invasive brachial blood pressures were 100/60mmHg and 115/50mmHg, respectively. Cardiac auscultation detected a murmur of a diastolic regurgitation at the right second intercostal space. The size of the abdomen was consistent with gestational age, foetal heart rate tracing was reassuring. A bedside Transthoracic echocardiography interpreted by a trained cardiologist in the department of cardiology revealed left ventricle with preserved ejection fraction, right ventricle with normal ejection fraction, moderate aortic regurgitation, a 6cm ascending thoracic aortic aneurysm, flap intimal in the aortic arch, absence of significant mitral valve disease, absence of pericardial effusion, and no pulmonary arterial hypertension. A thoracic computed tomography was performed urgently showed an image of the intimal flap with a circulating false channel starting from the aortic root and extending to the emergence of the left subclavian artery and dilation of the aortic orifice to 66.7mm and the ascending aorta to 50mm in favor of stanford type- a aortic dissection. Based on these findings, the patient was referred to the Cardiovascular surgery department for Bentall surgery and urgent cesarean delivery in coordination with the gynecologists.

**Figure 1:**
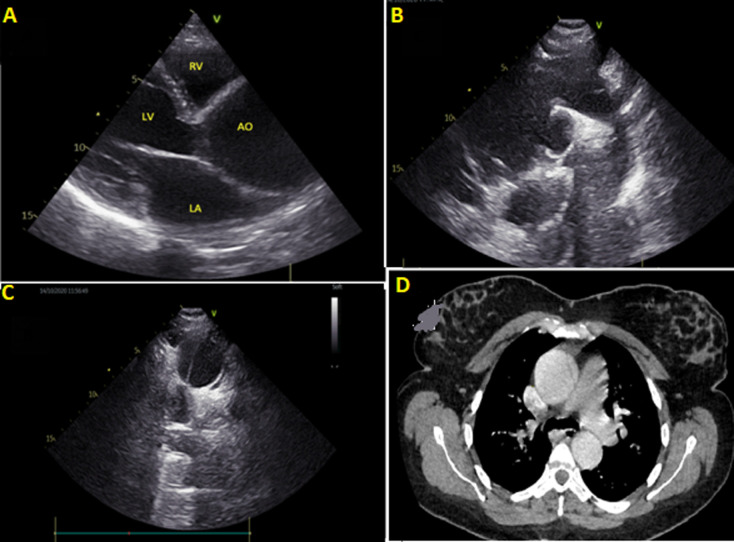
A) parasternal long axis of transthoracic echocardiography illustrating Annulo-aortic ectasia; B) parasternal short-axis view showing an intimal flap in the proximal ascending aorta; C) suprasternal view of aortic arch demonstrating enlargement of the ascending aorta; D) computed tomography angiography of the chest showing intimal flap in the proximal ascending aorta (axial view)

